# Recent Trends in Artificial Intelligence-Assisted Coronary Atherosclerotic Plaque Characterization

**DOI:** 10.3390/ijerph181910003

**Published:** 2021-09-23

**Authors:** Anjan Gudigar, Sneha Nayak, Jyothi Samanth, U Raghavendra, Ashwal A J, Prabal Datta Barua, Md Nazmul Hasan, Edward J. Ciaccio, Ru-San Tan, U. Rajendra Acharya

**Affiliations:** 1 Department of Instrumentation and Control Engineering, Manipal Institute of Technology, Manipal Academy of Higher Education, Manipal 576104, India; anjan.gudigar@manipal.edu (A.G.); sneha.nayak@manipal.edu (S.N.); 2 Department of Cardiovascular Technology, Manipal College of Health Professions, Manipal Academy of Higher Education, Manipal 576104, India; samanth.jyothi@manipal.edu; 3 Department of Cardiology, Kasturba Medical College, Manipal Academy of Higher Education, Manipal 576104, India; ashwal.aj@manipal.edu; 4 School of Management & Enterprise, University of Southern Queensland, Toowoomba, QLD 4350, Australia; Prabal.Barua@usq.edu.au; 5 Faculty of Engineering and Information Technology, University of Technology Sydney, Sydney, NSW 2007, Australia; 6 Department of Cardiology, Ad-Din Medical College Hospital, Dhaka 1217, Bangladesh; nazmulhasan45cmc@gmail.com; 7 Department of Medicine, Division of Cardiology, Columbia University Medical Center, New York, NY 10032, USA; edwardciaccio@gmail.com; 8 Department of Cardiology, National Heart Centre Singapore, Singapore 169609, Singapore; tan.ru.san@singhealth.com.sg; 9 Duke-NUS Medical School, Singapore 169857, Singapore; 10 School of Engineering, Ngee Ann Polytechnic, Clementi 599489, Singapore; Rajendra_Udyavara_ACHARYA@np.edu.sg; 11 Department of Biomedical Informatics and Medical Engineering, Asia University, Taichung 41354, Taiwan; 12 Department of Biomedical Engineering, School of Science and Technology, Singapore University of Social Sciences, Singapore S599494, Singapore

**Keywords:** artificial intelligence, computer aided diagnosis, coronary angiography, coronary artery disease, coronary computed tomographic angiography, intravascular optical coherence tomography, intravascular ultrasound

## Abstract

Coronary artery disease is a major cause of morbidity and mortality worldwide. Its underlying histopathology is the atherosclerotic plaque, which comprises lipid, fibrous and—when chronic—calcium components. Intravascular ultrasound (IVUS) and intravascular optical coherence tomography (IVOCT) performed during invasive coronary angiography are reference standards for characterizing the atherosclerotic plaque. Fine image spatial resolution attainable with contemporary coronary computed tomographic angiography (CCTA) has enabled noninvasive plaque assessment, including identifying features associated with vulnerable plaques known to presage acute coronary events. Manual interpretation of IVUS, IVOCT and CCTA images demands scarce physician expertise and high time cost. This has motivated recent research into and development of artificial intelligence (AI)-assisted methods for image processing, feature extraction, plaque identification and characterization. We performed parallel searches of the medical and technical literature from 1995 to 2021 focusing respectively on human plaque characterization using various imaging modalities and the use of AI-assisted computer aided diagnosis (CAD) to detect and classify atherosclerotic plaques, including their composition and the presence of high-risk features denoting vulnerable plaques. A total of 122 publications were selected for evaluation and the analysis was summarized in terms of data sources, methods—machine versus deep learning—and performance metrics. Trends in AI-assisted plaque characterization are detailed and prospective research challenges discussed. Future directions for the development of accurate and efficient CAD systems to characterize plaque noninvasively using CCTA are proposed.

## 1. Introduction

Coronary artery disease is a major cause of morbidity and mortality worldwide. Its underlying histopathology is the formation of atherosclerotic plaques within the wall lining of the coronary artery tree [1]. Chronic morphological adaptation of the plaque is characterized by progressive necrotic changes and calcification [2]. Initially, the plaque is nonobstructive and the patient is asymptomatic. Accumulation of plaque components increases the plaque atheroma volume, which encroaches on the coronary lumen [3,4]. As the coronary luminal area becomes reduced, blood flow delivery to heart muscles becomes compromised at high-demand stress states, i.e., myocardial ischemia, which manifests as angina [5]. Progressive arterial wall remodeling alters plaque composition and surface, rendering it vulnerable to erosion and even rupture [6]. This incites chain chemical reactions that precipitate acute thrombosis, which occludes the coronary lumen causing myocardial infarct [7]. 

Atherosclerotic plaques comprise heterogeneous components such as extracellular lipids, collagen fibers (fibrous tissue), loose collagen fibers with lipid accumulation (mixed tissue) and—when chronic—compact calcium crystal deposits (calcified tissue) [8]. The American Heart Association grades the atherosclerotic plaque into classes with incremental histo-morphological complexity [9]: Type I, normal wall thickness or minimal intimal thickening, some macrophages with little lipid deposits or foam cells; Type II, additional smooth muscle cells with little lipid deposits, T-lymphocytes and rare mast cells; Type III (pre-atheroma), increased extracellular lipid; Type IV, (atheroma), confluent lipid deposits (lipid core) covered mainly by intima; Type V, increasing lipid deposits with well-defined fibrous (collagen) cap (fibroatheroma, type Va) or predominant calcifications (calcific, types V b and c); Type VI, complicated lesion with disruption of lesion surface, hematoma or hemorrhage, and thrombotic deposits have developed [10]. 

Intravascular ultrasound (IVUS) and intravascular optical coherence tomography (IVOCT) performed via catheter during invasive coronary angiography (CAG) possess high spatial resolution and signal contrast, and are the reference standards for in vivo near-field characterization of individual atherosclerotic plaques. Based on the constituents, atheromatous plaques can be stratified as stable or vulnerable. The former is characterized by heavy calcification, fibrotic tissue, and small lipid pools. In contrast, the latter contains a large lipid pool (necrotic core) with or without spotty calcification, and is covered by a thin fibrous cap that is soft in nature and prone to rupture—the thin-cap fibroatheroma (TCFA). Post-processing techniques such as virtual histology intravascular ultrasound (VH-IVUS) [11] can enhance visualization of early pathology, e.g., intimal thickening (PIT), as well as high-risk lesions such as TCFA and calcified TCFA. Similarly, IVOCT can characterize human atherosclerotic plaques into fibrous, fibrocalcific and lipid rich types with histology-like accuracy [12]. Using IVOCT, TCFA was more frequently seen in patients presenting with acute coronary syndrome than those with stable angina [13].

Noninvasive coronary computed tomographic angiography (CCTA) can also characterize atherosclerotic plaques albeit with less fine spatial resolution than invasive IVUS and IVOCT. Of advantage, CCTA can survey the entire coronary tree within a single three-dimensional image dataset and possesses high sensitivity for calcium deposition. Coronary plaques can thus be categorized as noncalcified, partially calcified and calcified, and the severity of calcification quantitated. Using advanced analysis, lipid and fibrotic components in noncalcified plaques can also be identified [14].

Manual identification and characterization of coronary plaques demands clinical expertise and high time costs. It is also dependent on image quality, which is easily affected by speckle noise. These reasons motivate the development of computer aided diagnosis (CAD) systems for automated image processing as well as coronary plaque identification and characterization on both invasive [15] and noninvasive image readouts [16]. With the help of CAD, image quality can be improved, which helps in the accurate interpretation of results. Use of CAD by physicians can be considered as a countercheck to reduce diagnostic error and physician workload. Algorithms can enhance physician performance by increasing the ease and efficiency of decision-making. Given the importance of early diagnosis and risk stratification, accurate, and efficient automated diagnostic tools for coronary plaque characterization based on non-invasive imaging modalities like CCTA are desirable. In this review, our aims were as follows:

- To compare manual grading systems for plaque characterization with various imaging modalities;
- To analyze state-of-the-art artificial intelligence (AI) techniques to characterize plaque;
- To discuss the results and roles of different techniques for plaque characterization; and
- To highlight potential research gaps and future research directions related to plaque characterization using CAD.

The remainder of the paper is structured as follows: description of article selection process is in Section 2; review of commonly used imaging modalities for detecting and grading coronary plaques appears in Section 3; overview of different machine learning (ML) and deep learning (DL) techniques for coronary plaque detection and classification are in Section 4; comparison of the performance of various CAD techniques when applied to different imaging modalities as well as discussion of current challenges, potential research gaps, and future directions are found in Section 5; and study conclusions are in Section 6.

## 2. Review Process

The review process was carried out based on the procedures and guidelines presented in PRISMA [17]. We performed a search of the following databases, IEEEXplore, PubMed, Springer, Scopus, and Google Scholar, for articles published between 1 January 1995 and 25 June 2021 using the keywords ‘deep learning AND plaque detection’, ‘machine learning AND plaque detection’, ‘computer aided diagnostic tools’, ‘coronary plaque segmentation’, ‘computed tomography angiography’, ‘coronary plaque detection’, ‘intravascular ultrasound’, ‘intravascular optical coherence tomography’, ‘atherosclerotic plaque’, ‘plaque morphology’, ‘support vector machine AND plaque classification’ and ‘convolutional neural networks AND plaque detection’.

**Figure 1:**
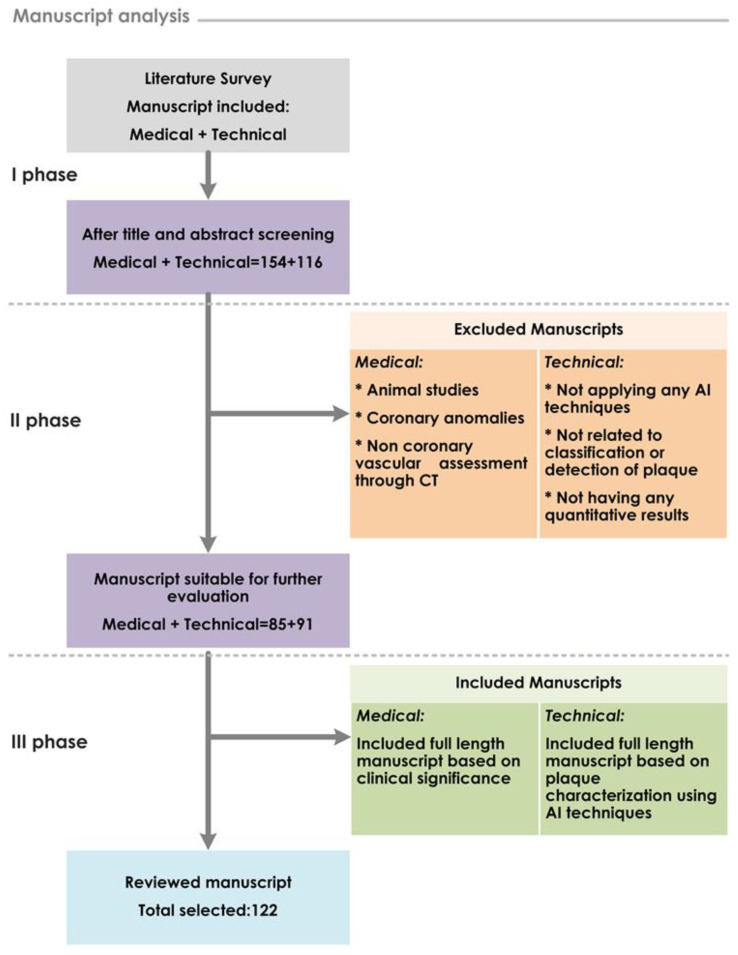
Workflow of study strategy and manuscript selection process.

To identify the most suitable and eligible articles: These articles were further filtered using inclusion/exclusion criteria. We included prospective studies that compared various imaging modalities such as CCTA, IVOCT and IVUS in the diagnosis and characterization of coronary atherosclerotic plaques. In addition, studies that employed various ML and DL to segment plaque regions from the image dataset, as well as to detect and classify coronary plaque type, were considered. We also considered clinical studies that focused on coronary plaque detection, grading, and characterization including coronary calcification. Studies related to image acquisition technique, non-coronary vascular assessment, coronary anomalies, coronary artery stenosis severity, and only animals were excluded. The selected articles comprised those with medical and technical contents (see Figure 1). The final selection of 61 medical and 61 technical publications from 154 and 116 screened, respectively, were all published in the last fifteen years.

## 3. Current Coronary Artery Disease Detection Modalities and Grading System

**Figure 2:**
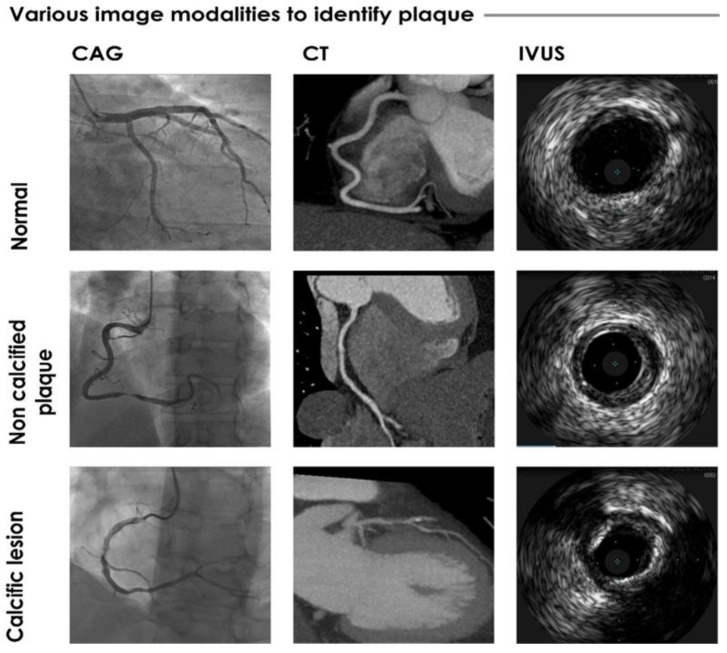
Sample images from invasive CAG, CT, and IVUS imaging techniques.

Several invasive and non-invasive modalities are employed in clinical practice for the identification and assessment of coronary plaques [18], e.g., IVUS (including VH-IVUS), IVOCT, CCTA (see Figure 2), positron emission tomography, magnetic resonance imaging, and near-infrared spectroscopy.

Invasive CAG is the gold standard for coronary artery disease diagnosis based on detection of coronary lumen stenosis but is of limited utility for plaque characterization beyond outlining intracoronary thrombus associated with ruptured atherosclerotic plaque. Nevertheless, invasive CAG is a prerequisite for plaque characterization with IVUS and IVOCT. IVUS is the gold standard for assessment of the atherosclerotic coronary plaque [19,20]. A miniaturized ultrasound probe is introduced intra-luminally via an intravascular catheter to the coronary lesion to obtain near-field two-dimensional cross-sectional high-resolution real-time images of the coronary lumen and atherosclerotic plaque with high echogenic grey-scale signal contrast [21,22]. IVUS is able to distinguish the intimal, medial, and adventitial layers of the arterial wall as well as various components of the atherosclerotic plaque, including the presence of dense calcium (DC), necrotic core (NC), fibrotic tissue (FT), and fibrofatty tissue (FFT) [23,24]. It yields comprehensive quantitative information on lumen size, plaque components and presence of vulnerable plaque features or complications, e.g., TCFA or plaque surface rupture, which herald an imminent acute coronary event [25]. VH-IVUS uses spectral analysis of the backscattered grey-scale IVUS radiofrequency signals to augment tissue contrast among the various plaque components [26]. The results are displayed in color codes, which enhances visualization of pathology. Instead of sound waves, IVOCT exploits light waves to produce near-field plaque images with superior spatial resolution (approximately 15 μm versus 150 μm for IVUS) and tissue discrimination but less imaging depth (few millimeters versus five to ten millimeters for IVUS), which may unveil surface details not appreciated on IVUS [27]. Calcified plaque identification as well as discrimination between fibrous and lipid tissues are better with IVOCT than with IVUS.

Multi-slice CCTA has evolved in the past two decades to become the dominant non-invasive imaging technique for coronary artery disease diagnosis [28,29], which provides information on coronary anatomy, calcification, location of stenosis and occlusion, as well as plaque morphology [8,9,10,30,31]. While CCTA image acquisition relies on ionizing radiation, the dose requirement has gradually been reduced by 30–50% through use of prospective electrocardiography-gated dose-dependent acquisition protocols on contemporary scanners [32]. While CCTA tends to overestimate coronary lesion severity compared with invasive CAG and IVUS [33], major trials that compared CCTA against quantitative coronary angiography with invasive CAG showed high sensitivity of 85–99% and fair specificity of 64–90% [34,35,36,37] for prediction of coronary artery stenosis. The clinical utility is highest in low-intermediate risk populations [38] rather than high-risk groups in which CAG with the option of therapeutic intervention at the same setting confers advantage. Calcified coronary plaques are readily visualized on computed tomography (CT) and the burden of coronary calcium, i.e., coronary calcium score, is an independent predictor of long-term outcome including incident death, myocardial infarction, and need for revascularization. However, the absence of coronary calcification does not imply the absence of coronary artery disease. Non-calcified plaques, significant coronary stenoses (50%), and >70% stenoses can be present in 11–13%, 0.9–3.7%, and 0.4–1.5% of patients with a coronary calcium score of zero [39,40,41].

Using early four-detector CT scanners, Becker et al. [42] showed that CCTA could effectively distinguish between histologically validated lipid-rich and fibrous-rich non-calcified plaques, with significant CT attenuation differences between the two. Technological advances in CT hardware (e.g., 256 or more multi-slice scanners), acquisition and post-processing have improved image resolution and the ability to detect plaques as well as characterize their composition and features, including high-risk non-calcified plaques [43,44,45,46,47,48,49]. CCTA has high positive predictive value (PPV) for identifying vulnerable plaques that are at risk for rupture [34,50]. While IVUS offers superior resolution compared with CCTA for quantitative plaque characterization [51,52,53], CCTA offers expedient comprehensive assessment of the entire coronary artery tree in a single acquisition, and is non-invasive. In a meta-analysis of 33 studies [54], CCTA demonstrated an excellent 90% sensitivity and 92% specificity prediction rate for coronary artery disease when referenced against IVUS.

## 4. Artificial Intelligence (AI): Characterization of Plaque

**Figure 3:**
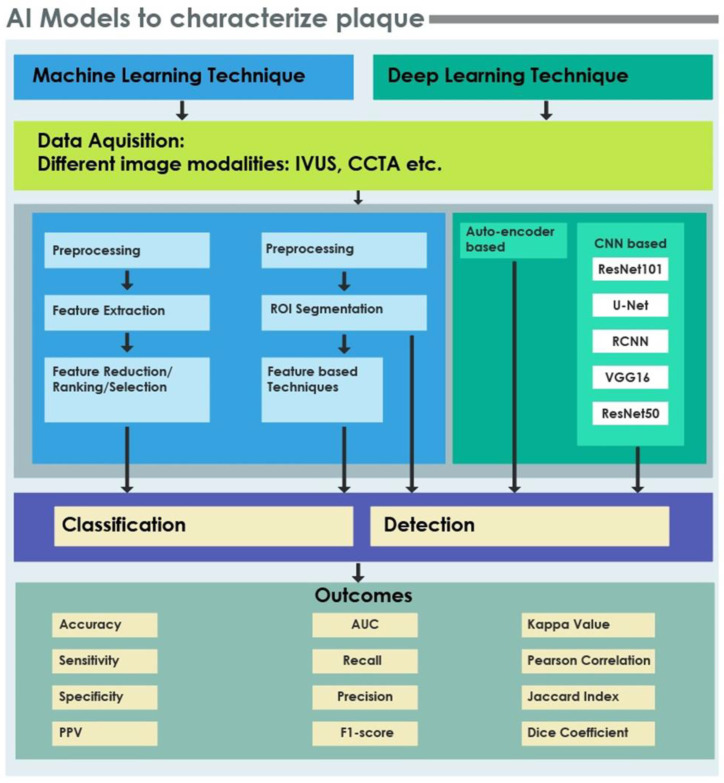
Processes, techniques and performance metrics of machine and deep learning models for coronary plaque characterization.

AI enables extraction and processing of quantitative information using human-like intelligence systems [55,56,57,58,59]. In recent years, the increase in generation and availability of medical imaging data has spawned research into clinical applications of AI [60,61,62,63,64,65]. Early detection of coronary plaque accumulation is important to avert complications, and many ML and DL CAD algorithms have been proposed for the automatic detection and classification of coronary plaques. CAD tools can improve clinical workflow efficiency by increasing the accuracy and timeliness of image interpretation. Figure 3 shows typical processes and techniques involved in ML and DL models for detection and classification of coronary plaques, as well as the standard performance metrics.

### 4.1. ML and DL Techniques in CAD

As described above, IVUS, IVOCT, and CCTA are the imaging modalities that can be used to characterize coronary atherosclerotic plaques. Research and development of CAD tools for plaque characterization have relied on image datasets from private [66,67,68] or public sources [69,70,71,72,73]. Some of the latter are available only upon request [25,72].

Machine Learning Techniques

ML uses various algorithms for the following key steps: preprocessing and segmentation; feature extraction; dimensionality reduction or feature ranking; and classification. Image preprocessing is a crucial preliminary step as it enhances the image quality or resolution using appropriate filters [74,75,76]. Extraction of the correct region of interest (ROI) is the necessary initial step for coronary plaque detection before further characterization. In order to detect or characterize the plaque, suitable features are generated using feature extraction algorithms and subsequently processed for dimensionality reduction. The extracted features may be ranked using feature ranking measures, which are then sent to a classifier to categorize the plaque type.

Deep Learning Techniques

Automated image recognition has improved tremendously since the widespread implementation of DL techniques, especially those with a convolutional neural network (CNN) architecture. CNN is composed of a series of layers, in which discriminative image features are extracted from raw data automatically [77,78]. In general, DL recreates complex patterns from the assembly of simple ones. The input image is fed into the deep CNNs at initialization, and the data is propagated through layers generally consisting of convolution, pooling, rectified linear unit, and fully connected layer [79].

#### 4.1.1. Preprocessing/Segmentation

Images obtained from different modalities may not be carry sufficient information for further processing or may be of low quality. Preprocessing helps to standardize image quality and increase image contrast. Denoising techniques include the anisotropic diffusion filter [80,81], Gaussian filter [82,83,84] and median filter. The adaptive histogram equalization technique can also be used to improve the image contrast. In order to remove interference, the Hough transform is used in [85]. The segmentation process is simplified by converting images to polar coordinates [86]. High intensity calcified plaques may also be normalized. Furthermore, Otsu’s thresholding method is applied [87]. In [88], the multilevel discrete wavelet frame decomposition is used for image segmentation. In [11], a hybrid fuzzy C-means and k nearest neighbor (HFCM-kNN) model is used for image segmentation. Other techniques include a region-growing method along with thresholding [89], and deformable models used for ROI extraction. The border can be delineated using k-means clustering and the Rayleigh mixture model [90]. Moreover, shadowed regions surrounding the plaque can be detected and removed using dynamic programming [91].

#### 4.1.2. Feature Extraction

To accurately detect the plaque, certain features are extracted from the image. Using the widely used gray level co-occurrence matrix (GLCM), textural features can be extracted based on the spatial relationship between pixels [92,93,94]. Haralick’s textural features exhibited a good discrimination for arterial wall and lumen identification [70]. In [95], the Gabor transform for different scales and angles along with six measurements of entropy were explored. Likewise, first order statistics (FOS) can be used for texture analysis [96]. To extract coronary lumen and plaque features, an adjacent pattern method was implemented. In addition, features are obtained using color moments of the histogram followed by statistical feature extraction [97]. Other authors have used an attenuation coefficient, which extracts features by selecting a window for each pixel so as to mark the pixular labels based on plaque type [84]. Different textural patterns exist in various coronary plaque components. Textural analysis methods such as neighboring gray level (NGL), local binary pattern (LBP) and modified run length (MRL) are suitable for distinguishing such plaque patterns. In order to differentiate the dense calcium tissues more significantly, fractal dimensions of the plaque components are computed using the box counting method. In addition, gray level run length matrix (GLRLM) and Law’s texture energy (LTE) are used to analyze the textural patterns in plaques. The various plaque components can be extracted using a multiresolution decomposition method, i.e., fast wavelet transform (FWT), by obtaining the frequency content of the images [98]. Generally, the plaque components are characterized by different intensity levels. Specifically, dense calcium is associated with the highest intensity components. Extracting intensity of the plaque components improves the classification accuracy. Properties such as individual calcification levels, image brightness, and contrast, are obtained using an intensity histogram (IH). The neighborhood gray tone difference matrix (NGTDM), gray level difference statistics (GLDS), and invariant moment (IM) are incorporated to extract plaque textural features [99]. In [73], a multi-class coronary plaque detection framework random radius symmetry (RRS) containing contextual features of plaque was proposed that supplemented the training data of coronary plaques. Likewise, local indicators of spatial association (LISA) and run length (RL) are used to describe the textural features by detection. Several other algorithms have also been proposed for feature extraction, such as open lumen border tracing (OLBT), closed lumen border tracing (CLBT), extracting confluent components (ECC), and plaque burden assessment (PBA) [11].

#### 4.1.3. Feature Reduction/Selection/Ranking/Organization

Several factors influence the success of classification algorithms in any given task. For instance, the degree of redundant information reduces the efficiency of plaque classification. In [88], a fuzzy complementary criterion (FuzCoC) was used to select the features for plaque component discrimination. To identify the best extracted features, the *t*-test or wrapped feature selection (WRP) [83] can be used. In [69], among feature selection techniques such as Relief-F, recursive feature elimination, and the Fisher method, the latter achieved the highest accuracy for ranking feature discriminative power. Further, principal component analysis (PCA) and genetic algorithms (GA) can be used to select the optimal feature set from the original feature set for tissue characterization. Informative features obtained using PCA often contain small values, which are normalized using the Z-score function [100]. In [95], feature reduction was performed using locality sensitive discriminant analysis (LSDA) and neighborhood preserving embedding (NPE), and the results were compared with no feature reduction on the dataset. The latter test generated the most discriminant features. Thereafter, the generated features were selected using the analysis of variance (ANOVA) statistic. The Wilcoxon signed-rank test can also be used for feature organization [101].

#### 4.1.4. Classification

Plaques can be effectively categorized as calcified and non-calcified plaques using the Bayesian classifier [80]. In [92], an overall classification accuracy of 80.41% was achieved using the random forest (RF) classifier, in which the plaque was categorized into four types, namely calcium, lipid pool, fibrous tissue, and mixed plaques. Moreover, RF classifiers can classify the plaque into three classes, namely dense calcium (DC), necrotic core (NC), and fibrotic tissue (FT) and fibro-fatty tissue (FFT). Support vector machines (SVM) [73] are effectively used for coronary plaque characterization in most studies that utilize different kernel functions. SVM with a second order polynomial kernel function produced better performance parameters for carotid and coronary plaque characterization [99]. Error correcting output codes (ECOC) are the preferred medium for multiclass plaque classification [91]. For the discrimination of dense calcium tissue, the deep belief network model offers improved plaque characterization performance. Further, the atheromatous plaque can be characterized by a neuro fuzzy classifier. The FaIRLiC, hybrid ensemble classifier (feed forward neural network (FFNN), RF, adaptive boosting) can classify coronary plaque regions with high accuracy [94,98].

For plaque classification, deep learning models such as ResNet50-v2 and DenseNet-121 from ImageNet are used for binary as well as multiclass plaque classifications [102]. Plaques may be of varying severity. Based on their severity, they may be categorized as mild, moderate, or severe using a SVM-based CNN. Moreover, stenosis can be detected using U-Net and V-net [103]. Table 1 summarizes these various classification methodologies.

In [112], calcified regions are identified by detecting the acoustic shadow and analyzing the ROI extracted for the presence or absence of calcification in the IVUS images. The results of the proposed algorithm showed high correlation with human expert measurements. Likewise, a semiautomatic method for segmentation and quantification of calcified plaques was applied on IVOCT images based on a level-set model approach [87]. Along with quantified analysis of calcification, its boundary could be detected [113]. Detection of calcified plaque in the presence of acoustic shadowing is quite challenging. A Markov random field and graph searching algorithm was implemented in [90]. Along with calcified plaque detection, the lumen was detected using the K-means algorithm [82]. The calcified plaque could be determined based only on its 3D position, independent of the volume and shape of the plaque, using a blob enhancing filter [89]. However, coronary plaque composition, plaque position, and length of the plaque in abnormal coronary segments were better determined using the mean radial profile and SVM [69]. Soft and hard plaque detection can be ascertained using Fuzzy C-means (FCM) clustering, morphological processing, and a curve fitting function, which showed a high comparison coefficient compared with a manual plaque detection system. In addition to plaque detection, plaque shape and size can be quantitated [86]. Similarly, vulnerable plaques can be identified using a flexible neural tree (FNT) [81].

CNN-based DL techniques are commonly used for plaque classification or identification. A distribution-preserving autoencoder-based neural network (DPAE–NN) implemented on IVOCT images was found to be best suited for characterization of heterogeneous plaques [114]. Images passed through the ResNet101 architecture with fc1000 as the output layer and a naïve Bayesian (NB) classifier outperformed other methods for calcified plaque detection [115]. In [116], high sensitivity was obtained for calcification detection using Inception-ResNet-v2 along with a naïve Bayes classifier. Similarly, CNN architectures ResNet-50, ResNet-101 and Inception-v3 using SVM and the discriminant analysis (DA) classifier could characterize plaques more efficiently. In [101], the 3D CNN model, along with a SegNet architecture, was utilized for calcified plaque segmentation. In addition to plaque detection, the 3D U–Net CNN was used in challenging tasks such as coronary artery lumen delineation and stenosis grading [79]. The DL technique termed recurrent convolutional neural network (RCNN) detects coronary artery stenosis by extracting the coronary artery centerline during a skeletonization process [108,109]. Lumen and artery wall media detection, which is crucial in identifying plaque buildup in the walls of coronary vessels, can be achieved with a CNN (U-Net + VGG16 encoder) [71]. Coronary calcium assessment, or calcium score, is an important predictor of cardiovascular events. Calcium scores are predicted using a CNN (U-Net) in [117,118]. Table 2 summarizes the various detection methodologies described.

## 5. Discussion

In this study, we present a systematic catalogue of CAD using ML and DL techniques for coronary atherosclerotic plaque characterization using various imaging modalities.

### 5.1. Role of Various Modalities in Coronary Artery Disease

When referenced to invasive CAG, CCTA has sensitivity of 73–99%, specificity of 54–94%, positive predictive value of 64–92% and negative predictive value (NPV) of 83–100% for coronary artery disease detection [36,125,126,127,128,129,130,131]. For detection of plaque in acute coronary syndrome, CCTA has good sensitivity and NPV (100% and 100%, respectively) [129]. Two meta-analyses, comprising 40 studies that compared CCTA with CAG and 41 diagnostic accuracy studies along with five prognostic studies, respectively, showed excellent pooled sensitivity of 99% and median NPV of 100% [132]. Similarly, a meta-analysis of nine studies showed a high sensitivity of 96% (95% CI: 93% to 98%), specificity of 86% (95% CI: 83% to 89%), positive likelihood ratio of 6.38 (95% CI: 5.18 to 7.87) and negative likelihood ratio of 0.06 (95% CI: 0.03 to 0.10) for CCTA against the reference standard CAG [28]. For detecting coronary artery disease in the low-to-intermediate risk group, the diagnostic accuracy improved with more advanced CT scanner models as well as with use of CT-based fractional flow reserve (FFR). CT FFR is derived using computational fluid dynamics simulation of the 3D anatomic coronary artery tree model, and is able to inform on the physiological significance of individual coronary artery stenoses. Using contrast gradient attenuation along an arterial lesion, CCTA has high diagnostic accuracy (ROC AUC of 0.88, 95% CI 0.81–0.96, *p* < 0.001) and sensitivity, specificity, PPV and NPV of 77%, 74%, 67% and 86%, respectively, when referenced against invasive FFR-assessed coronary artery ischemia [133]. CT-based FFR showed a high specificity of 88% for coronary artery disease detection in [134]. Two meta-analyses involving studies that compared CCTA with invasive FFR showed good diagnostic accuracy of CCTA for the detection of functionally significant stenoses with pooled sensitivity of 92% and 84.6%, and NPV of 84.6% and 87.3%, respectively [135,136]. Detection of plaque characteristics through CCTA had a sensitivity and specificity greater than 90% compared to IVUS according to two meta-analyses involving 75 studies in total [137]. Detection of plaques with a napkin ring sign, which denote unstable plaque, by CCTA had a sensitivity of 93.8% and NPV of 66.7% when referenced against invasive IVUS findings [129]. Overall, the literature supports the use of CCTA for noninvasive diagnostic assessment of coronary artery disease.

### 5.2. Role of CAD in Coronary Artery Disease

Automatic coronary plaque detection has been studied in 61 manuscripts included in this review report: 16 manuscripts are based on CCTA, 28 based on IVUS, 16 manuscripts are based on IVOCT, and one is based on X-ray angiography.

Most studies have implemented classification techniques to categorize image data sets as either containing plaque, no plaque, or mixed plaque. Different classifiers were used and the performance metrics of the classifiers reported in terms of outcomes, such as sensitivity, specificity, AUC, accuracy, PPV, and NPV. The majority of articles reported sensitivity (30 manuscripts), specificity (24 manuscripts) and accuracy (38 manuscripts) as performance metrics. Performance parameters such as AUC, PPV, and average precision were also used to assess performance. Among the classifiers, SVM was found to outperform, and was implemented in 14 articles [120,124]. The RF classifier [105] and FaIRLiC classifier were used in some studies. A novel approach using a hybrid classifier containing FFNN, RF, and AdaBoost was implemented by some authors. Some works focused on plaque detection, i.e., to determine the presence or absence of plaque. CNN was the most preferred model for plaque detection (implemented in 23 studies) [104,106,107,110,119,121,123].

In this review, performance outcomes for plaque detection and classification using ML and DL were compared. In [90], the highest sensitivity of 94.68% was reported for detection of calcified plaque with acoustic shadowing using a Markov random field and graph searching algorithm, based on the evaluation of 996 in vivo IVUS images acquired from eight patients. Compared with IVOCT, CCTA showed a high sensitivity of 93.2% when tested on 32 datasets [69]. A high specificity of 98.5% was achieved for automated segmentation of calcified plaque using a Bayesian classifier for a dataset that comprised 60 IVUS images from seven patients [80]. IVUS outperformed other modalities with an average accuracy of 98.9% for the classification of atheromatous plaque, which confirmed its clinical utility in plaque characterization [15]. Furthermore, a PPV of 96.69% was achieved in coronary and carotid plaque characterization using the SVM classifier implemented on 2685 IVUS images obtained from 15 patients [99]. 100% sensitivity, specificity, and accuracy were obtained using a DL approach for detecting calcification in IVUS images in a dataset that comprised 2175 images acquired from ten patients. The results were obtained using the ResNet-50, ResNet-101 and Inception-v3 CNN architecture with SVM classifier and discriminant analysis classifier [2]. Based on the aforementioned findings, IVUS outperforms the IVOCT and CCTA. CCTA demonstrated better results when compared with IVOCT, with an accuracy of 99% using CNN [117]. Studies showed that, by using the CNN ResNet101 architecture for feature extraction and a naïve Bayesian classifier, a PPV of 100%, accuracy of 99.95%, sensitivity of 99.81%, and specificity 100% were achieved in for detecting calcification for IVUS images acquired from ten patients [115]. ImageNet using models ResNet50-v2 and DenseNet121 was used for binary and multiclass plaque classification in the dataset comprising 4000 images acquired from 49 patients, in which accuracy of 91.7%, specificity of 92.4% and sensitivity of 90.9% were reported [102].

**Figure 4:**
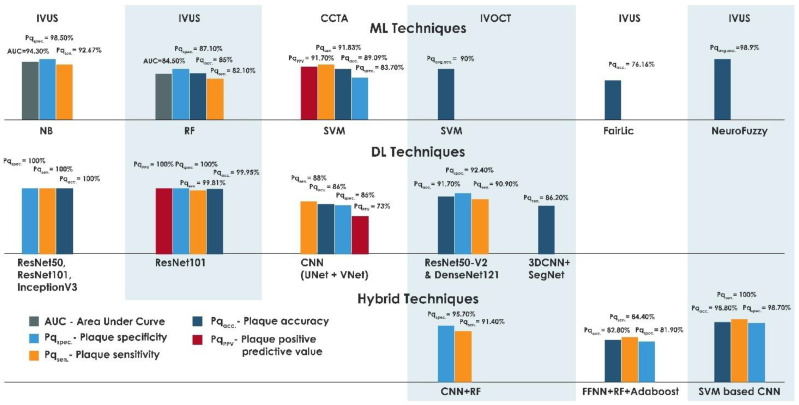
Plot of best-performing techniques with various modalities.

Supervised learning and a DL algorithm were combined to form a hybrid method SVM-based CNN algorithm, which effectively characterized the image pixels into plaque and non-plaque regions. This hybrid model achieved an accuracy of 98.8%, sensitivity of 100%, and specificity of 98.70% on IVUS images acquired from seven patients [97]. Similarly, CNN and RF were implemented on the IVOCT images of 49 patients for binary plaque classification, yielding classification sensitivities of 84.8% and 91.4%, and specificities of 97.8% and 95.7%, respectively, for fibro-lipidic and fibrocalcific plaques [72]. A hybrid novel image-based classification with an ensemble classifier consisting of FFNN, RF and AdaBoost showed an increased accuracy of 82.8% compared with other single classifiers [98]. Figure 4 depicts the performance of all the methods.

### 5.3. Research Opportunities and Future Direction

It is observed that many studies in this field are based on IVUS. While invasive techniques are the reference standards for identifying and grading plaques and are helpful for understanding the underlying etiology of cerebrovascular disease, CCTA has emerged as the dominant noninvasive modality for studying coronary atherosclerosis. With CCTA, the identification of vulnerable plaques and their quantification relies on expert interpretation and is dependent on the degree of the interobserver variability. In addition, CCTA cannot supplant invasive techniques for more subtle plaque characteristics such as erosion and neovascularization [31]. While 3D reconstruction of plaque using CT slices would alert the physician to the presence of coronary artery disease at an early stage, it is advantageous that the investigator concentrates on hybrid feature extraction techniques when characterizing plaques using CAD based on CCTA images. There are challenges involved in the development of efficient CAD to characterize plaque [138,139,140]. These are discussed in detail below.

*Availability of plaque datasets*: Currently there is a lack of datasets that are publicly available. Most of the CAD are developed based on private datasets with low sample numbers. Hence, it is difficult to generalize from such analyses. Another challenge is to develop a large dataset with annotated plaque severity. This process is tedious and expensive, as it requires the input of expert radiologists. Data imbalance with various imaging modalities and their levels of severity may cause overfit or underfit of the training models. In order to handle skewed data, augmentation can be utilized in all modalities. The collection of more data of the different modalities can be achieved with the help of collaborative partner hospitals and clinics.

*Comparative study on CAD using various modalities*: There is a dearth of studies that have compared different modalities for the development of CAD to characterize plaques. Hence, a major challenge for researchers is to develop an efficient CAD to identify plaques using various imaging modalities, such as CCTA and IVUS images of the same patient. Investigators should concentrate on obtaining an automatic grading system using noninvasive imaging technique such as CCTA. This should then be compared with the expert grading system to produce a generalized CAD for plaque categorization. Finally, the CAD should be fast, noninvasive, inexpensive, and accurate, so that it can be used anywhere to obtain plaque deposition results without the need for expert involvement.

*CAD in the assessment of prognosis*: Though several CAD are developed to detect and characterize plaques, none of the studies have reported on prognosis as well as responsiveness to treatment. Hybrid methods, such as a combination of ML and DL based approaches, can be further explored to develop CAD that correlates initial plaque deposition before and after treatment, as well as with long-term clinical outcome.

**Figure 5:**
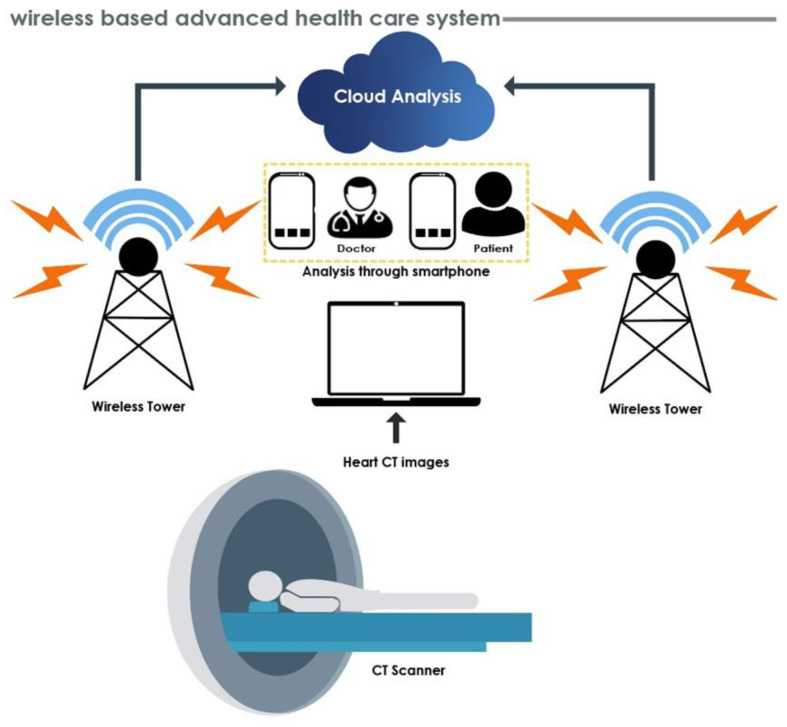
Future healthcare system using wireless networks.

Generally, physicians or radiologists assess plaque deposition and its grading by using predefined image frames on IVUS, IVOCT and CCTA, and the results may vary from expert to expert based on their expertise in the various modalities, hence delaying treatment. Therefore, the diagnostic assessment of patients is typically performed by tertiary hospitals that are equipped to obtain and interpret the required images. It is thus difficult for geographically separated patients to obtain high-level diagnosis and treatment in a timely manner. In the future, cloud-based wireless healthcare systems can provide improved diagnostics (please refer to Figure 5). These consist of cloud-based methodologies, wherein hybrid CAD are available online to process the data. The CT scanner to obtain heart CT images noninvasively is connected to the cloud through a wireless network. Finally, CT images are processed and results sent to doctors and patients via mobile phone. This enables remote coordination of care by doctors of patients suspected of having coronary artery disease. This will assist in obtaining an overall goal of providing inexpensive yet high quality diagnostics in the rural areas of developing countries.

### 5.4. Limitations of the Study

Some limitations of the study are given below:

1. The present review has been carried out based on manuscripts written in English. Other language manuscripts were not included during the review process;
2. The current review process included a plaque grading system using various modalities, and analysis of various AI algorithms to develop CAD for plaque categorization. However, review on grading during plaque deposition and after treatment was not given substantial consideration;
3. The specific reasons to select the algorithms based on AI were not mentioned. It was also unclear whether the proposed CAD can improve the survival of patients.

## 6. Conclusions

This review summarizes both medical and technical manuscripts for plaque characterization. A total of 61 medical manuscripts were used to understand the manual plaque grading schemes with various modalities and 61 technical papers were analyzed to understand the application of AI algorithms in the prediction of plaque deposition, classification, and detection. These methods were summarized and discussed along with various aspects of research challenges. Experimental results show that AI algorithms using ML and DL based methods have merits for identifying plaques, and can be used as a valuable resource in the medical decision-making process. In the future, these AI methods can be exploited to achieve better results by addressing future challenges and implementing the AI models in real clinical trials.

## Figures and Tables

**Table 1:** Summary of various state-of-the-art techniques employed for plaque characterization using coronary artery scans.

	Dataset	Preprocessing/ROI Segmentation	Feature Extraction	Feature Reduction/Feature Selection/ Feature Ranking/Organization	Detection	Classification	Task	Outcomes *
[11]	599 VH-IVUS images of 10 patients	Thresholding + HFCM-kNN model	CLBT + OLBT			SVM with radial basis function (RBF)	Multiclass (PIT, TCFA and CaTCFA) and binary (TCFA and non-TCFA)	For binary: Pq_acc._: 81.03 Pq_sen._: 84.81 Pq_spec._: 84.81 Precision: 84.81 For multiclass Pq_avg.acc._: 98.42 + 0.01 Kappa: 0.9198
[15]	IVUS images					Neuro Fuzzy	Atheromatous plaque (fibrotic, lipidic, calcified, and normal)	Pq_avg.acc._: 98.9
[21]	300 IVUS frames of 10 patients	Deformable models + Estimation borders by experts	Co-occurrence matrix + LBP+ Mean value + Entropy + Geometrical features	*t*-test		RF	Multiclass (DC, NC, FT, and FFT)	Pq_acc._: 85.65
[22]	553 IVUS frames of eight patients	ROI Extraction + Otsu’s automatic thresholding + Pathological tissue detection	CNN				Multiclass (DC, NC, FT, FFT, Media)	Overall accuracy: 93.5Pqacc.:DC: 98.5NC: 88.6FT: 91.1FFT: 90.0Media: 99.4
[25]	IVUS images from 11 patients	Manual segmentation by expert	LBP + FOS +GLCM + LEM + Extended GLRLM + Intensity	PCA		RF	Multiclass(DC, NC, FT, and FFT)	AUC: 0.845, 0.704, 0.783 Pq_acc._: 85.1,71.9,77.2 Pq_sen._: 82,81.2, 80.6 Pq_spec._: 87.1, 59.6, 75.9 (Respectively for Net1: FT/FFT or NC/DC Net2: FT or FFT Net3: NC or DC)
[27]	1000 IVOCT images from 47 patients	Anisotropic diffusion + Polar Transformation+ Hough Transform	Intensity + HOG + LBP + FV + *k*-means clustering			SVM	Multiclass (normal, fibrous plaque, fibro-atheroma, plaque rupture, fibro-calcific plaque)	Pq_avg.acc._: 90 With standard deviation of 0.02
[67]	770 OCT images of 5 patients	ROI Extraction	LBP+GLCM	CNN (U Net)			Multiclass (lipid tissue, fibrous tissue, background)	Pq_acc._: 95.8
[70]	435 IVUS images	Polar Transformation + Gaussian filter +Median filter+ Anisotropic Diffusion filter	Haralick’s +Laws’ textural feature			SVM	Two class (fibrotic plaque and normal)	AUC: 0.97 Jaccard Index: 0.85
[72]	6556 OCT images from 49 patients	ROI+ Dynamic programming + Gaussian filter	CNN + Morphological features	Wilcoxon signed rank test		RF	Binary class: (fibro-lipidic and fibrocalcific plaque)	Fibro-lipidic plaque: Pq_sen._: 84.8 Pq_spec._: 97.8 Fibro-calcific plaque: Pq_sen._: 91.4 Pq_spec._: 95.7
[73]	18 CTA images Total: 1786 cross sections with Non calcified plaque (NCP): 729, Calcified plaque (CP): 511, Mixed plaques: 546.	DRLSE	RRS feature vector			SVM (Gaussian kernel)	Multiclass(calcified, non calcified and mixed plaques)	Average precision: 92.6±1.9 Average recall: 94.3±2.1
[80]	60 IVUS images of 7 patients	Anisotropic diffusion filter + Thresholding			Deformable models	Bayesian	Two class (calcified and non-calcified plaque)	AUC: 0.943 Pq_spec._: 98.5 Pq_sen._: 92.67
[83]	27 OCT pullbacks of 22 patients	Gaussian filter + Thresholding + *k*-means	LBP + GLCM	WRP		RF	Multiclass (calcium, lipid pool, fibrous tissue, and mixed Tissue)	Pearson’s correlation coefficient: 0.97 (FT)
[84]	IVOCT images of 11 patients	Gaussian filter + Otsu threshold filtering	Attenuation coefficient + GLCM			SVM (RBF)	Multiclass (fibrous, calcification and lipid tissue)	Pq_acc._: 83
[88]	IVUS images of 7 patients	Multilevel discrete wavelet frame decomposition	FOS + GLCM + LBP + RL + Wavelet Intensity values	FuzCoC		SVM (RBF)	Multiclass (calcium, necrotic core, fibrous, and fibro-fatty)	Pq_avg.acc._: 81
[91]	In-vivo dataset: VH-IVUS 2263 images of 10 patients Ex-vivo dataset:64 images	Shadow detection using threshold	NGL + LBP + MRL			SVM and ECOC	Multiclass(calcium, necrotic core, and fibro fatty)	Kappa values: 0.639 (in-vivo) and 0.628 (ex-vivo)
[92]	50 OCT images from 3 patients		Co-occurrence matrix + LBP+ Entropy + Mean value			RF	Multiclass (calcium, lipid pool, fibrous tissue, and mixed plaque)	Pq_acc._: 80.41
[93]	300 IVUS images of 7 patients	Multilevel discrete wavelet frames decomposition + SOFM	FOS + GLCM + RL + LBP + wavelets + LISA			FaIRLiC	Multiclass (DC, NC, FT, and FFT)	Testing accuracy: 76.16%
[94]	IVUS images of 7 patients	Border detection + 2-D Kohonen’s self-organizing feature map (SOFM)	FOS + GLCM + WF + RL + LBP			FaIRLiC	Multiclass(calcium, necrotic core, fibrous and fibro lipid)	Average classification Accuracy on each frame: 73.67
[95]	2646 Coronary Tomography Angiography (CTA) images of 73 patients (CP: 28, NCP: 15, Normal: 30)	Adaptive Histogram Equalization	Gabor Transform + Entropy	ANOVA		SVM (RBF and polynomial kernel)	Multiclass (normal, non calcified and calcified)	Pq_acc._: 89.09 Pq_PPV_: 91.70 Pq_sen._: 91.83 Pq_spec._: 83.70
[96]	316 IVUS images of 26 patients	Thresholding + Polar transformation + Morphological operations	FOS + FD (Box counting) + GLCM + GLRLM + LTE	PCA		Deep belief network	Multiclass (DC, NC, FT, and FFT)	Pq_sen._: 92.8 ± 0.1, Pq_spec._: 85.1 ± 0.1, Pq_acc._: 88.4 ± 0.1, Pq_PPV_: 86 ± 0.1 Pq_NPV_: 91.2 ± 0.1 (*p* < 0.05).
[97]	IVUS images of 7 patients		Adjacent pattern algorithm + Color moments of histogram + Statistical features	SVM based CNN			Multiclass (mild, moderate and severe)	Pq_acc._: 98.80, 98.80, 97.59 Pq_sen._: 100, 100, 100 Pq_spec._: 98.70, 98.70, 97.40 Precision: 85.71, 85.71, 75 Recall: 100, 100, 100 F-score: 0.92, 0.92, 0.99 (Respectively for Mild, moderate and severe)
[98]	IVUS images from 11 patients	Manual border segmentation	FOS + GLCM + GLRLM + LBP + Intensity + Discrete wavelet features +LTE	Genetic algorithm		Hybrid ensemble classifier(FFNN+ RF+ Ada boost)	Multiclass (DC, NC, FT, and FFT)	Pq_acc._: 82.8, 71.6, 77 AUC: 0.832, 0.697, 0.787 Pq_sen._: 84.4, 81.9, 74.9 Pq_spec._: 81.9, 57.6, 82.4 Pq_PPV_: 71.2, 72.4, 91.7 Pq_NPV_: 90.8, 70.1, 55.9 (Respectively for Net1: FT/FFT or NC/DC Net2: FT or FFT Net3: NC or DC)
[99]	2685 IVUS images of 15 patients	ImgTracer software	GLCM + GLRLM + IH + GLDS + NGTDM + IM + Statistical feature matrix			SVM (polynomial kernel 2nd order)	Coronary and carotid plaque	Pq_acc._: 94.95 AUC: 0.95 Pq_sen._: 92.88 Pq_spec._: 96.61 Pq_PPV_: 96.69
[100]	588 VH-IVUS images of 10 patients	Fuzzy c means and *k* means with particle swarm optimization	LBP + GLCM + MRL	PCA		SVM (RBF)	TCFA and Non-TCFA	Pq_acc._: 98.61
[102]	4000 IVOCT images from 49 patients	Cartesian Transformation	CNN from ImageNet ResNet50-v2 and DenseNet-121				Binary class: plaque (calcified plaque and lipid/fibrous plaque) and no plaque	Pq_acc_.: 91.7 Pq_sen._: 90.9 Pq_spec._: 92.4
[103]	CCTA of 150 patients	CNN (U Net + V Net)					Stenosis Detection and Plaque classification (calcified, partially calcified, noncalcified and no plaque)	Stenosis identification: CCTA AI (p<0.001) AUC: 0.870 Pq_acc._: 86 Pq_sen._: 88 Pq_spec._: 85 Pq_PPV_: 73 Pq_NPV_: 94 Plaque classification: AUC: 0.750
[104]	12,325 IVUS images from 100 patients	IVUS and OCT registration + ROI segmentation	CNN				Binary (thin cap fibro-atherma and normal)	AUC: 0.911 Pq_spec._: 82.81 Pq_sen._: 87.31
[105]	64 IVOCT images from 49 patients	Otsu’s method + morphological operation	Attenuation + Texture			RF	Multiclass (fibrotic, calcified, and lipid rich)	Pq_acc._: 81.5
[106]	4469 IOCT images of 48 patients	Edge detection + Gaussian filter		*t*-test	CNN followed by post processing (Conditional Random Field + Morphological processing)		Multiclass (fibrocalcific, fibro-lipidic and other classes)	Pq_acc._: 77.7 ± 4.1, 86.5 ± 2.3, 85.3 ± 2.5 Pq_sen._: 80, 85, 84 Pq_spec._: 95, 92, 92 (Respectively for fibrocalcific, fibro-lipidic, other classes) *p*-value: 0.00027
[107]	700 OCT images of 28 patients					CNN	Multiclass (calcium, lipid tissue, fibrous tissue, mixed tissue, media and no visible tissue)	Pq_acc._: 96
[108]	CCTA scans of 131 patients	3D Recurrent Convolutional Neural Network					Multiclass (no plaque, non-calcified, mixed, calcified) and stenosis (no stenosis, non- significant, significant)	Plaque analysis: Pq_acc._: 72, F1 score: 0.61 Cohen’s kappa: 0.60 Stenosis analysis: Pq_acc._: 81 F1 score: 0.78 Cohen’s kappa: 0.70 Pq_sen._: 61 Pq_PPV_: 83
[109]	CCTA scans of 163 patients	Recurrent convolutional neural network					Multiclass(calcified, non-calcified and mixed)	Plaque detection: Pq_acc._: 77 F1 score: 0.61 Cohen’s kappa: 0.61 Stenosis detection: Accuracy: 80% F1 score: 0.75 Cohen’s kappa: 0.68 Pq_sen._: 61 Pq_PPV_: 65
[110]	CTA scans from 25 patients	Multiplanar reformation technique	3D CNN U-Net (Encoder-decoder)				Multiclass (calcified plaque, non-calcified plaque and mixed calcified plaque)	Dice scores: 0.83, 0.73, 0.68 Pq_sen._: 85, 76,72 Pq_PPV_: 82, 69, 62 Respectively for CAP, NCAP, MCAP
[111]	2060 CTA images from 60 patients		Higher-order spectra cumulants	Multiple factor analysis + *t*-test		SVM(RBF)	Binary (calcified, noncalcified)	Pq_acc._: 95.83 Pq_sen._: 94.54 Pq_spec._: 97.13 Pq_PPV_: 97.05

* AUC: Area Under Curve, Pq_PPV_ (%): Plaque Positive Predictive Value, Pq_NPV_ (%): Plaque Negative Predictive Value, Pq_sen._ (%): Plaque Sensitivity, Pq_spec._ (%): Plaque Specificity, Pq_acc._ (%): Plaque accuracy, Pq_avg.acc_. (%): Plaque average accuracy, Precision (%).

**Table 2:** Summary of various state-of-the-art techniques employed for plaque characterization using coronary artery scans.

	Dataset	Preprocessing/ROI Segmentation	Feature Extraction	Feature Reduction/Feature Selection/ Feature Ranking/Organization	Detection	Classification	Task	Outcome *
[2]	2175 IVUS images of 10 patients 530 images with calcification 1645 images without calcification	Original image resized and converted to RGB	ResNet50, ResNet101, Inception-v3			SVM and DA	Calcified plaque detection	Pq_acc._: 100 Pq_sen._: 100 Pq_spec._: 100
[66]	CT images of 56 patients	Thresholding		CNN (ConvNet)			Calcification identification	Pq_sen._: 91.24 Pq_spec._: 95.37 Pq_PPV_: 90.5 Pearson coefficient: 0.983 Cohen’s kappa: 0.879
[68]	CCTA of 493 patients	Centerline extraction + Clamping technique				3D CNN	Atherosclerosis detection	Pq_avg.acc._: 90.9 Pq_PPV_: 58.8 Pq_sen._: 68.9 Pq_spec._: 93.6 Pq_NPV_: 96.1 Average AUC: 0.91
[69]	32 datasets of CTA	Normalization of high intensity calcified plaque		Fisher method	Mean radial profile	SVM Gaussian RBF	Soft plaque detection	Pq_acc._: 88.4 Pq_sen._: 93.2 Pq_spec._: 80.3 Dice coefficient: 0.832
[71]	435 IVUS scan images	CNN (U-Net + VGG16 encoder)					Detection of lumen and media	For media: Avg Jaccard measure: 0.8085 Avg Dice score: 0.8825 For Lumen: Avg Jaccard measure:0.7982 Avg Dice score:.8846
[79]	78 CCTA images of 18 patients	3D U-Net CNN					Coronary artery lumen segmentation (for grading stenosis)	Dice: 0.8291
[81]	1000 OCT images	Polar Transformation + Anisotropic diffusion			Flexible neural tree		Vulnerable plaque detection	Pq_acc._: 90.80
[82]	27 OCT images from 10 patients	Gaussian filter + Thresholding			*k*-means clustering		Calcified plaque detection	Pq_sen._: 83 Pq_PPV_: 74 Pearson correlation: 0.434
[85]	1000 OCT images	Hough Transform + Polar transformation	CNN				Fibrous plaque detection	Pq_acc._: 94.12 Recall: 94.12
[86]	60 IVUS images from 7 patients	Polar Transformation			GLCM + FCM + ROI selection + Morphological processing + Curve fitting		Detection (Hard plaque and soft plaque)	Pq_spec._: 83 Pq_sen._: 91
[87]	106 IOCT images of 8 patients	Otsu’s thresholding + Edge detection			Intensity + level-set model		Segmentation of calcified plaque	Pq_acc._: 78 ± 9
[89]	CCTA of 7 patients	Thresholding + 3D region growing			Blob enhancing filter		Stenosis by Calcified plaque	Precision:94.4
[90]	996 in-vivo IVUS images of 8 patients	Rayleigh mixture model			Markov random field and Graph searching algorithm		Calcified plaque detection	Pq_sen._: 94.68 Pq_spec._: 95.82
[101]	8231 IOCT images of 68 patients	Dynamic programming + semantic segmentation method + Gaussian filter		Wilcoxon signed rank test	3D CNN + SegNet		Calcified plaque segmentation	Pq_sen._: 86.2 Precision: 75.8 F1 score: 0.781
[112]	20 IVUS images	Adaptive thresholding			Priori information of the acoustic Shadow		Calcification detection	Pq_spec._: 88 Pq_sen._: 84 AUC: 0.87
[113]	2175 IVUS images of 10 patients	Otsu thresholding + Morphological operation + Empirical threshold					Detection of calcification boundary	Pq_acc._: 82 Pq_sen._: 80 Pq_spec._: 84 Pq_PPV_: 83
[114]	30 OCT images	DPAE–NN					Binary (detection of plaque and normal tissues)	AUC: 0.9132 Pq_acc._: 93.6 Kappa score: 0.62
[115]	2175 IVUS image of 10 patients with 530 calcified images and 1645 without calcification	Original image resized and converted into RGB	CNN architecture ResNet101			NB	Calcification detection	Pq_acc._: 99.95 Pq_sen._: 99.81 Pq_spec._: 100 Pq_PPV_: 100 Pq_NPV_: 99.94
[116]	2175 IVUS images of 10 patients Calcified images: 530 Noncalcified: 1645	Images resized and converted to RGB			Inception-ResNet-v2	NB	Calcification detection	Pq_sen._: 100 Pq_spec._: 95.87 Pq_PPV_: 88.63 Pq_acc._: 96.87 AUC: 0.9967
[117]	903 CT scans	ConvNet					Calcium scoring	Cohen’s kappa: 0.95 Precision:77 Recall: 85 Pq_acc._: 99 Dice score: 0.81
[118]	CT scans of 20084 individuals	CNN (U-Net)					Assessment of calcium score	AUC: 0.74
[119]	8914 IVUS images of 80 patients	CNN					Calcified plaque detection	Average F1 score: 0.67 Average precision: 77 Average recall: 83
[120]	105 IVUS pullback dataset	Polar transformation				SVM (RBF)	Calcified plaque detection	Pq_acc._ > 90 Precision: 96 Recall: 93
[121]	713 grayscale IVUS images of 18 patients	CNN U-Net architecture					Detection (media adventitia, lumen and calcium regions)	Average precision: 73 Pq_sen._: 72 Pq_spec._: 99 Mean Dice score function (DSC): 0.67Spearman’s correlation: 0.92
[122]	CTA of 12 patients	Thresholding + Difference of Gaussian filter (DOG)			Fuzzy c means + Median filter		Calcified plaque detection	Recall: 94 Precision: 94
[123]	2D axial CTA images of 50 patients	U-Net			CNN-RNN model	CNN	Segmentation of coronary artery (stenosis detection)	Recall:95.9 Precision: 97.9 Pq_acc._: 96.1
[124]	CTA images of 30 patients	Hessian matrix+ Thresholding+ Morphological operations				SVM	Stenosis detection	Average Recall: 94.08 Precision: 88.59

* AUC: Area Under Curve, Pq_PPV_ (%): Plaque Positive Predictive Value, Pq_NPV_(%): Plaque Negative Predictive Value, Pq_sen._ (%): Plaque Sensitivity, Pq_spec._ (%): Plaque Specificity, Pq_acc._ (%): Plaque accuracy, Pq_avg.acc._ (%): Plaque average accuracy, Precision (%).

## Data Availability

Not applicable.
